# Impact of Sustained Virological Response for Gastroesophageal Varices in Hepatitis-C-Virus-Related Liver Cirrhosis

**DOI:** 10.3390/jcm9010095

**Published:** 2019-12-30

**Authors:** Yukihisa Yuri, Hiroki Nishikawa, Hirayuki Enomoto, Kazunori Yoh, Yoshinori Iwata, Yoshiyuki Sakai, Kyohei Kishino, Naoto Ikeda, Tomoyuki Takashima, Nobuhiro Aizawa, Ryo Takata, Kunihiro Hasegawa, Noriko Ishii, Takashi Nishimura, Hiroko Iijima, Shuhei Nishiguchi

**Affiliations:** 1 Division of Hepatobiliary and Pancreatic disease, Department of Internal Medicine, Hyogo College of Medicine, Nishinomiya, Hyogo 663-8501, Japan; gyma27ijo04td@gmail.com (Y.Y.); enomoto@hyo-med.ac.jp (H.E.); mm2wintwin@ybb.ne.jp (K.Y.); yo-iwata@hyo-med.ac.jp (Y.I.); sakai429@hyo-med.ac.jp (Y.S.); hcm.kyohei@gmail.com (K.K.); nikeneko@hyo-med.ac.jp (N.I.); tomo0204@yahoo.co.jp (T.T.); nobu23hiro@yahoo.co.jp (N.A.); chano_chano_rt@yahoo.co.jp (R.T.); hiro.red1230@gmail.com (K.H.); ishinori1985@yahoo.co.jp (N.I.); tk-nishimura@hyo-med.ac.jp (T.N.); hiroko-i@hyo-med.ac.jp (H.I.); nishiguc@hyo-med.ac.jp (S.N.); 2 Center for clinical research and education, Hyogo College of Medicine, Nishinomiya, Hyogo 663-8501, Japan

**Keywords:** liver cirrhosis, hepatitis C virus, gastroesophageal varices, sustained virological response, interferon, direct-acting antivirals

## Abstract

We aimed to clarify the relationship between sustained virological response (SVR) and gastroesophageal varices (GEVs) progression among hepatitis C virus (HCV)-related liver cirrhosis (LC) patients treated with interferon (IFN)-based therapies (*n* = 18) and direct-acting antiviral (DAA)-based therapies (*n* = 37), and LC patients with no SVR (*n* = 71) who had already developed GEVs. Factors influencing GEVs progression were also examined. During the follow-up period, GEVs progression was observed in 50 patients (39.7%). The 3-year cumulative GEVs progression rates in the DAA-SVR group, the IFN-SVR group, and the non-SVR group were 32.27%, 5.88%, and 33.76%, respectively (overall *p* value = 0.0108). Multivariate analysis revealed that sex (*p* = 0.0430), esophageal varices (EVs) F2 or more (*p* < 0.0001), and DAA-SVR (*p* = 0.0126, IFN-SVR as a reference) and non-SVR (*p* = 0.0012, IFN-SVR as a reference) were independent predictors for GEVs progression. The proportion of GEVs progression in patients with no or F1 EVs was significantly lower than that in patients with F2 or F3 EVs (33.9% (38/112) vs. 85.7% (12/14), *p* = 0.0003). In conclusion, IFN-based therapies can have a favorable impact for preventing GEVs progression in HCV-related LC patients with GEVs. Clinicians should be aware of a point of no return where SVR is no longer capable of avoiding GEVs progression.

## 1. Introduction

Chronic hepatitis C virus (HCV) infection is one of the major causes of morbidity and mortality worldwide, mainly due to complications of liver cirrhosis (LC), portal hypertension (PH), and hepatocellular carcinoma (HCC) [1,2]. HCV eradication (i.e., sustained virological response (SVR)) in patients with chronic HCV infection implies substantial changes in numerous aspects, including liver histological findings, liver function, incidence of HCC, muscle mass, and quality of life [3,4,5,6,7,8,9,10]. These favorable clinical outcomes are independent of treatment regimens used to achieve SVR and have been well acknowledged since the advent of interferon (IFN)-based therapies [11]. However, in LC patients with gastroesophageal varices (GEVs), IFN-based therapies have been shown to result in lower SVR rates due to the risk of severe adverse events, despite the presence of GEVs being a significant predictor for survival [11,12,13]. Therefore, their applicability was limited for the data collection in this population. IFN-free, direct-acting antivirals (DAAs) are not subject to these limitations in difficult-to-treat patient populations [8,14,15,16,17,18,19]. Magina et al. reported that LC patients with PH receiving sofosbuvir and velpatasvir pangenotypic combination therapy can achieve SVR comparable to those of patients without PH [15]. The introduction of DAAs has dramatically changed treatment efficacy in HCV-related LC patients with GEVs. DAAs have thus opened a new door for such patients.

Afdhal et al. reported that chronic HCV and compensated or decompensated LC patients with SVR can have clinically meaningful decreases in hepatic venous pressure gradient (HVPG), which is the best surrogate marker for PH, at long-term follow-up [20]. In contrast, some LC patients maintain clinically significant PH (CSPH) (HVPG 10 mmHg or more) even after SVR [21,22,23]. However, analyses of SVR after DAA treatment in LC patients with GEVs have not yet been fully performed and might provide further insights to help with the individualization of the treatment for LC patients. A point of no return might exist where HCV eradication is no longer capable of averting the progression of PH or liver decompensation [24]. In addition, data for the comparison of the impact of HCV eradication on GEVs progression among LC patients treated with IFN-based therapies or DAA-based therapies, and LC patients with non-SVR (persistent viremia) are currently scarce. Addressing these clinical research questions may be highly relevant for a better understanding of SVR in LC patients with GEVs. The aim of this study was thus to clarify the relationship between SVR and GEVs progression among HCV-related LC patients treated with IFN-based therapies, DAA-based therapies, and LC patients with non-SVR who had already developed GEVs. 

## 2. Patients and Methods

### 2.1. Patients 

Between June 2005 and August 2018, a total of 126 HCV-related LC individuals with GEVs and follow-up data for existing GEVs by esophagogastroduodenoscopy (EGD) were admitted to our hospital (Hyogo College of Medicine Hospital, Japan). All patients had no clear evidence of hepatitis B virus coinfection or HIV coinfection. All patients were instructed to refrain from alcohol intake. HCV RNA levels were tested using a Roche COBAS Taq Man test. HCV genotype was tested by polymerase chain reaction (PCR) amplification of the core region of the HCV genome using genotype-specific PCR primers [25]. Patients were considered to have achieved SVR if they remained negative for serum HCV RNA 24 weeks after the end of antiviral therapy [25]. Out of the 126 patients analyzed, 18 received IFN-based therapies and SVR was reached (the IFN group), 37 received DAA-based therapies and SVR was reached (the DAA group), and the remaining 71 did not reach SVR during the follow-up period (the non-SVR group). Some patients with no past history for IFN-based therapies (persistent viremia without antiviral therapies) were included in the non-SVR group. In decompensated LC patients, antiviral therapies were performed after full consideration of potential treatment-related adverse events. In all analyzed subjects, GEVs were confirmed by EGD. 

### 2.2. GEVs and Our Study Endpoint

GEVs were classified into three groups (i.e., F1, F2, or F3) by expert endoscopists based on the current guidelines [26]. In our department, endoscopic therapies for GEVs are considered in patients with F2 varices and positivity for red color sign, or F3 varices on EGD. In principle, GEVs were assessed every 6–12 months on EGD. Our primary study endpoint was GEVs progression on GED (enlargement of existing GEVs or varices rupture). We retrospectively investigated the relationship between SVR and GEVs progression among LC patients treated with IFN-based therapies and DAA-based therapies, and LC patients with no SVR who had already developed GEVs. Factors influencing on GEVs progression were examined using uni- and multivariate analyses. Written informed consent was obtained from all analyzed subjects. The ethical committee of our hospital approved the current study protocol (approval no. 3096 and 3317) and this study strictly followed all regulations of the Declaration of Helsinki. During data collection, personal data were protected.

### 2.3. Definition of Follow-Up

In the DAA group and the IFN group, follow-up period was defined as the time interval from the date of the EGD prior to commencement DAA- or IFN-based therapies to the first confirmation of GEVs progression on EGD or last follow-up EGD. In the non-SVR group, follow-up period was defined as the time interval from the first confirmation of GEVs on EGD to the first confirmation of GEVs progression on EGD or the last follow-up EGD without additional antiviral therapies. 

### 2.4. Statistical Analyses

Quantitative variables were compared by unpaired *t*-test, Mann–Whitney *U* test, Analysis of variance (ANOVA) or Kruskal–Wallis test, as applicable. Categorical variables were compared by Fisher’s exact test or Pearson’s χ^2^ test, as applicable. Continuous variables were divided into two groups at each median value, which were then treated as dichotomous variables for univariate analysis. Curves for cumulative GEVs progression were created using the Kaplan–Meier method and compared by the log-rank test. Parameters with a *p* value < 0.05 in the univariate analysis were subject to the multivariate analysis in the Cox proportional hazard model. Data are presented as the median value (interquartile range (IQR)) unless otherwise indicated. *p* values < 0.05 were considered to suggest statistical significance. Statistical analysis was performed with JMP Pro 14 software (SAS Institute Inc., Cary, NC, USA).

## 3. Results

### 3.1. Baseline Characteristics

The baseline characteristics in this study (*n* = 126) were presented in Table 1. The study included 70 males and 56 females with a median age (IQR) of 66 (61.75, 70.25) years. A Child–Pugh score of A was in the majority (82/126, 65.1%). HCV genotype 1 was in the majority (97/126, 77.0%). HCV high viral load (5 log IU/mL or more) was in the majority (101/126, 80.2%). Ascites and encephalopathy were identified at baseline in 28 (22.2%) and 8 patients (6.3%), respectively. The median (IQR) follow-up period was 2.84 (1.21, 4.66) years. In terms of the severity of GEVs, F3, F2, and F1 esophageal varices (EVs) were observed in 0, 14 and 107 patients, and F3, F2, and F1 gastric varices (GVs) were observed in 1, 10, and 34 patients. The median interval from the date when EGD was done for the confirmation of GEVs to the start of antiviral therapies were 123 days in the IFN group and 127 days in the DAA group. 

### 3.2. Treatment for SVR

In the IFN-SVR group, pegylated (PEG)-IFN plus ribavirin combination therapy was done in 11 patients, IFN plus ribavirin combination therapy in 3 patients, and IFN monotherapy in 4 patients. In the DAA-SVR group, daclatasvir plus asunaprevir combination therapy was done in 16 patients, sofosbuvir plus ledipasvir combination therapy in 13, and other therapies in 8. 

### 3.3. GEVs Progression

During the follow-up period, GEVs progression was observed in 50 patients (39.7%, progression of EVs in 48 patients and progression of GVs in 2 patients). In the IFN group, GEVs progression was found in 5 patients (27.8%), in 10 (27.0%) in the DAA group, and in 35 (49.3%) in the non-SVR group. Of these, varices rupture was found in nine patients and one patient died due to varices rupture. In cases with GEVs progression, appropriate therapies were chosen through discussion with gastroenterologists and radiologists considering the current guidelines [26,27]. 

### 3.4. Comparison of Baseline Characteristics among IFN-SVR, DAA-SVR, and Non-SVR Groups

Baseline data in the IFN-SVR group, the DAA-SVR group, and the non-SVR group are shown in Table 2. Factors with significant overall *p* values among three groups were prothrombin time (*p* = 0.0018) and platelet count (*p* = 0.0002). 

### 3.5. Cumulative GEVs Progression Rates for the Entire Cohort 

**Figure 1:**
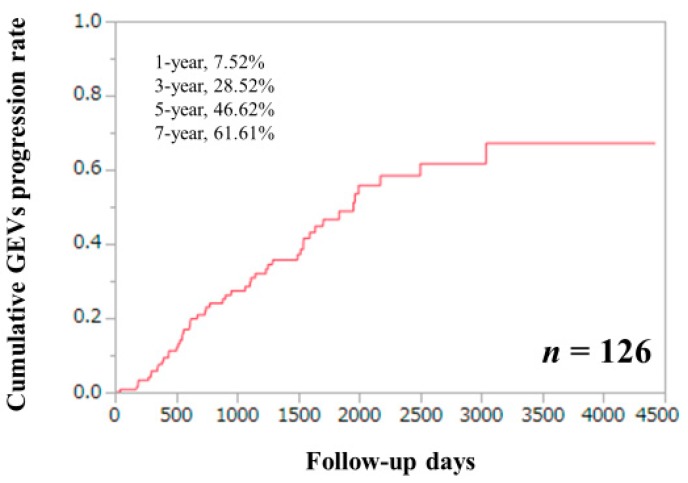
Cumulative GEVs progression rate for all cases (*n* = 126).

**Figure 2:**
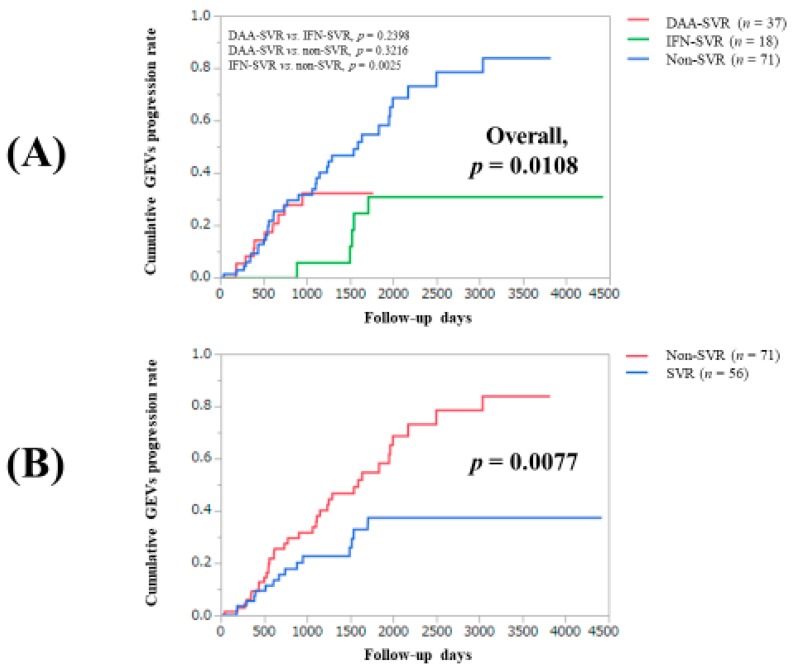
(**A**) Cumulative GEVs progression rates in the DAA-SVR group, the IFN-SVR group, and the non-SVR group. (**B**) Cumulative GEVs progression rates in the SVR group (DAA-SVR and IFN-SVR) and the non-SVR group.

For the entire cohort, the 1-, 3-, 5-, and 7-year cumulative GEVs progression rates were 7.52%, 28.52%, 46.62%, and 61.61%, respectively (Figure 1). In the DAA-SVR group, the 1- and 3-year cumulative GEVs progression rates were 8.19% and 32.27%, while in the IFN-SVR group and non-SVR group, the 1-, 3-, 5-, and 7-year cumulative GEVs progression rates were 0%, 5.88%, 30.98%, and 30.98% in the IFN-SVR group, and 9.2%, 33.76%, 45.36%, and 78.47% in the non-SVR group, respectively (*p* values: *p* = 0.2398, the IFN-SVR group vs. the DAA-SVR group; *p* = 0.3216, the non-SVR group vs. the DAA-SVR group; *p* = 0.0025, the non-SVR group vs. the IFN-SVR group; overall *p* value, *p* = 0.0108). (Figure 2A) The difference of cumulative GEVs progression rates between patients with SVR (the IFN-SVR group and the DAA-SVR group, *n* = 55) and the non-SVR group reached significance (*p* = 0.0077) (Figure 2B).

### 3.6. Univariate and Multivariate Analyses of Parameters Contributing to GEVs Progression

Univariate analysis found the following parameters to be significantly associated with GEVs progression: sex (*p* = 0.0253), our type classification for SVR (*p* = 0.0108), and EVs of F2 or more (*p* < 0.0001) (Table 3). The hazard ratios (HRs) and 95% confidence intervals (CIs) calculated by multivariate analysis for the three significant parameters (*p* < 0.05) in the univariate analysis are shown in Table 4. Sex (*p* = 0.0430), F2 or higher EVs (*p* < 0.0001), and DAA-SVR (*p* = 0.0126, IFN-SVR as a reference) and non-SVR (*p* = 0.0012, IFN-SVR as a reference) were independent predictors for the GEVs progression.

### 3.7. Child–Pugh Score and GEVs Progression Rate between Patients with EVs F1 or None and Those with EVs F2 or F3

**Figure 3:**
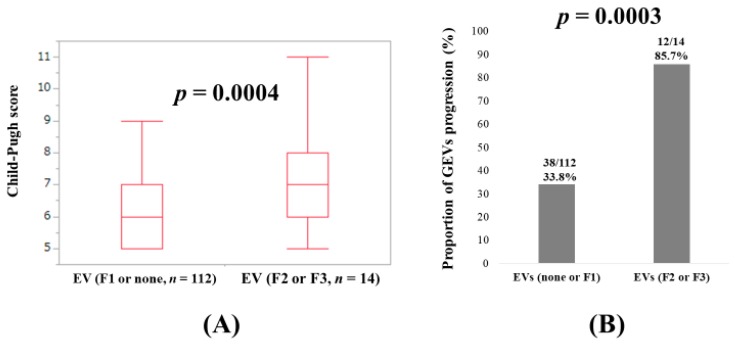
(**A**) Child–Pugh score in baseline no or F1-graded EVs and EVs graded F2 or F3. (**B**) Proportion of GEVs progression in baseline no or F1-graded EVs and EVs graded F2 or F3.

Because EVs graded F2 or more had the highest HR (5.898) for GEVs progression in the multivariate analysis, we further examined the relationship between Child–Pugh score and EV grade. In patients with no or F1-graded EVs (*n* = 112) and those with EVs graded F2 or F3 (*n* = 14), the difference of Child–Pugh score reached significance (*p* = 0.0004) (Figure 3A). The proportion of GEVs progression in patients with no or F1-graded EVs was significantly lower than that in patients with EVs graded F2 or F3 (33.9% (38/112) vs. 85.7% (12/14), *p* = 0.0003) (Figure 3B).

## 4. Discussion

To the best of our knowledge, this is the first study of its kind to compare the impact of SVR on GEVs progression among LC patients treated with IFN-based therapies and DAA-based therapies, and LC patients with no SVR who had already developed GEVs. The introduction of oral DAA agents led to dramatic improvement of SVR rates in HCV therapy, providing SVR rates of more than 95% with shortened HCV treatment duration and a good safety profile [28,29,30]. However, the impact of SVR treated with DAA-based therapies compared to patients receiving IFN-based therapies with SVR or patients with no SVR remains unclear. IFN-based therapies for HCV patients are less common these days; however, the questions addressed in our current research may be clinically meaningful.

In our data, the cumulative GEVs progression rates were well stratified in patients with or without SVR, and in the multivariate analysis, DAA-SVR (HR = 4.496, IFN-SVR as a reference) and non-SVR (HR = 5.126, IFN-SVR as a reference) were negative predictors linked to GEVs progression. These results denoted that IFN-SVR can contribute to reducing GEVs progression in HCV-related LC patients who have already GEVs. In the direct comparison between the IFN-SVR group and the DAA-SVR group, the difference in the two groups regarding GEVs progression rates did not reach significance (*p* = 0.2398). These discrepancies can be partly explained by the small number of cases in the IFN-SVR group (*n* = 18). On the other hand, it is noteworthy that GEVs progression was found in 5 patients (27.8%) in the IFN group, and in 10 (27.0%) in the DAA group in our results. The similar percentage between the two groups (27.8% vs. 27.0%) could be explained by the difference of the observation periods in the two groups. Additionally, a recent study reported that among 50 (33.1%) out of 151 (33.1%) LC patients with baseline low-risk GEVs receiving DAAs, 12 (24%) developed high-risk GEVs [22]. These data could be associated with the fact that CSPH can persist in most LC patients even after SVR [23].

In our results, EVs graded F2 or more had the highest HR (5.898) for GEVs progression in the multivariate analysis. Additionally, in patients with no or F1-graded EVs and those with EVs graded F2 or F3, statistical significance was observed in Child–Pugh score (*p* = 0.0004), and the proportion of GEVs progression in patients with none or F1 and EVs graded F2 or F3 reached significance (*p* = 0.0003). These results suggest that SVR cannot suppress GEVs progression when it falls into the far advanced LC group (i.e., decompensated LC). Bruno et al. reported that SVR can prevent the development of EV in patients with compensated HCV-induced LC (*n* = 218) in the long term follow-up period (median, 11.4 years) in the era of IFN therapies [31]. A previous prospective study also showed that SVR was associated with a lower incidence of EVs in compensated LC patients treated with PEG-IFN and ribavirin combination therapy (HR = 0.23, 95% CI: 0.11–0.48), and HCV eradication reduced the risk for liver decompensation regardless of whether the patients had EVs [8]. In another study (104 LC patients with PH receiving DAAs), Child–Pugh B patients were less likely to have a HVPG reduction (HR = 0.103, *p* = 0.006), when compared to Child–Pugh A patients [32]. Considering these reports and our results, a point of no return where HCV elimination is no longer capable of suppressing progression of PH or liver decompensation possibly exists, as mentioned in our introduction section. PH may be less likely to show improvement in decompensated LC patients after antiviral therapies [32]. When we looked at our Kaplan–Meier curves in the IFN group and the DAA group, we found that the Kaplan–Meier curves became flat after a certain time point, whereas in the non-SVR group, such tendencies were not observed. Improvement of liver fibrosis obtained by SVR may be linked to these observations [17,33,34].

In this study, the DAA-SVR group included 40.5% (15/37) of patients classified as Child–Pugh B, while the IFN-SVR group included only 22.2% (4/18). These results were also confirmed by a significantly lower platelet count and prothrombin time in the DAA-SVR group. A higher risk of progression in the DAA-SVR group could be influenced by the worse basal clinical condition of this group of patients. Indeed, Child–Pugh B patients may be less likely to present HVPG reduction, and subsequent GEVs progression may be more likely [32]. 

Several limitations must be acknowledged in our current analysis. First, this study was a single-center, retrospective observational study. Second, the number of patients in the IFN group and the DAA group was small for analysis. Third, during the observation period, various interventional or nutritional therapies such as diuretics or branched-chain amino acid supplementation for underlying LC were performed in each patient, potentially leading to bias. Fourthly, the long-term outcome of GEVs in the DAA group is unknown due to the relatively shorter follow up period in the DAA group, as shown in our Kaplan–Meier curves. We thus believe that there is urgent need to clarify the long-term outcomes of GEVs in LC patients treated with DAAs.

## 5. Conclusions

We conclude that IFN-based therapies can have a favorable impact for preventing GEVs progression in HCV-related LC patients who have already GEVs. In addition, clinicians should be aware of the presence of a point of no return where SVR is no longer capable of avoiding GEVs progression.

## Figures and Tables

**Table 1:** Baseline characteristics (*n* = 126).

Variables	All Cases (*n* = 126)
Age (years)	66 (61.75, 70.25)
Sex, male/female	70/56
HCV genotype, 1/2/others/not tested	97/19/6/4
HCV viral load, high/low	101/25
Child–Pugh classification, A/B/C	82/43/1
Our type classification	18/37/71
IFN-SVR/DAA-SVR/non-SVR	
Presence of ascites, yes/no	28/98
Presence of encephalopathy, yes/no	8/118
Total bilirubin (mg/dL)	1.0 (0.7, 1.4)
Serum albumin (g/dL)	3.7 (3.3, 4.0)
Prothrombin time (%)	76.2 (69.175, 84.3)
Platelet count (×10^4^/mm^3^)	7.7 (6.0, 9.925)
AST (IU/L)	60.5 (41.75, 87.25)
ALT (IU/L)	49 (30, 82.25)
Endoscopic findings	
Esophageal varices, F3/F2/F1/not detected	0/14/107/5
Gastric varices, F3/F2/F1/not detected	1/10/34/81

Data are expressed as median value (interquartile range). HCV: hepatitis C virus; IFN: interferon; SVR: sustained virological response; DAA: direct-acting antiviral; AST: aspartate aminotransferase; ALT: alanine aminotransferase.

**Table 2:** Baseline characteristics in the IFN-SVR group, the DAA-SVR group, and the non-SVR group.

Variables	IFN-SVR (*n* = 18)	DAA-SVR (*n* = 37)	Non-SVR (*n* = 71)	*p* Value IFN vs. DAA	*p* Value IFN vs. Non	*p* Value DAA vs. Non	Overall *p* Value
Age (years)	66.5 (57.75, 70)	68 (63, 70)	66 (62, 71)	0.2996	0.5088	0.5218	0.5648
Sex, male/female	13/5	16/21	41/30	0.0510	0.2944	0.1622	0.1089
Child–Pugh A/B/C	14/4/0	22/15/0	46/24/1	0.2343	0.5420	0.6261	0.6274
Ascites, yes/no	4/14	6/31	18/53	0.7128	0.9999	0.3358	0.5558
Encephalopathy, yes/no	0/18	2/35	6/65	0.9999	0.3409	0.7126	0.4059
Total bilirubin (mg/dL)	0.9 (0.7, 1.325)	1.1 (0.8, 1.35)	1.0 (0.7, 1.5)	0.1130	0.3126	0.5245	0.3545
Serum albumin (g/dL)	3.8 (3.375, 4.125)	3.5 (3.3, 3.9)	3.8 (3.3, 4.0)	0.2161	0.4688	0.6302	0.5706
Prothrombin time (%)	78.15 (66.175, 86.225)	71.4 (66.05, 76.45)	78.8 (71.2, 87.0)	0.0455	0.6464	0.0004	0.0018
Platelet (×10^4^/mm^3^)	10.45 (8.8, 14.075)	7.7 (5.6, 10.55)	7.2 (5.8, 9.2)	0.0151	<0.0001	0.1682	0.0002
AST (IU/L)	52 (33, 79)	53 (41.5, 79.5)	63 (43, 93)	0.6996	0.9956	0.1931	0.2961
ALT (IU/L)	48 (26.25, 99.75)	40 (28.5, 76)	54 (32, 85)	0.5300	0.8142	0.0993	0.1346
EVs, F3/F2/F1/ND	0/1/16/1	0/4/32/1	0/9/59/3	0.7236	0.6833	0.8800	0.9088
GVs, F3/F2/ F1/ND	0/3/7/8	0/2/11/24	1/5/16/49	0.2384	0.2059	0.7561	0.4466

Data are expressed as median value (interquartile range). IFN: interferon; SVR: sustained virological response; DAA: direct-acting antiviral; AST: aspartate aminotransferase; ALT: alanine aminotransferase; EVs: esophageal varices; GVs: gastric varices; ND: not detected.

**Table 3:** Univariate analyses of factors linked to esophageal or gastric varices progression (*n* = 126).

Variables	Number of Each Category	Univariate
		*p* Value
Age (years) 66 or more, yes/no	69/57	0.5812
Sex, male/female	70/56	0.0253
Presence of ascites, yes/no	28/98	0.2331
Presence of encephalopathy, yes/no	8/118	0.1877
Child–Pugh A, yes/no	82/44	0.2607
Our type classification, IFN-SVR/DAA-SVR/non-SVR	18/37/71	0.0108
Esophageal varices F2 or more, yes/no	14/112	<0.0001
Gastric varices F2 or more, yes/no	11/115	0.5475
Serum albumin 3.7 g/dL or more, yes/no	66/60	0.4992
Total bilirubin 1.0 mg/dL or more, yes/no	71/55	0.7782
Prothrombin time 76.2% or more, yes/no	63/63	0.0821
Platelet count 7.7 ×10^4^/mm^3^ or more, yes/no	64/62	0.3308
AST 60.5 IU/L or more, yes/no	63/63	0.1204
ALT 49 IU/L or more, yes/no	64/62	0.5389

IFN: interferon; SVR: sustained virological response; DAA: direct-acting antiviral; AST: aspartate aminotransferase; ALT: alanine aminotransferase.

**Table 4:** Multivariate analyses of factors linked to esophageal or gastric varices progression.

Variables	Multivariate Analysis		
	Hazard Ratio	95% CI	*p* Value
**Sex**			
Male	Reference		
Female	0.524	0.280–0.980	0.0430
**Esophageal varices F2 or more**			
No	Reference		
Yes	5.898	2.900–11.995	<0.0001
**Our type classification**			
IFN-SVR	Reference		
DAA-SVR	4.496	1.380–14.655	0.0126
Non-SVR	5.126	1.910–13.756	0.0012

CI: confidence interval; IFN: interferon; SVR: sustained virological response; DAA: direct-acting antiviral.

## Abbreviations

HCV	hepatitis C virus
LC	liver cirrhosis
PH	portal hypertension
HCC	hepatocellular carcinoma
SVR	sustained virological response
IFN	interferon
GEVs	gastroesophageal varices
DAAs	direct-acting antivirals
HVPG	hepatic venous pressure gradient
CSPH	clinically significant portal hypertension
EGD	esophagogastroduodenoscopy
PCR	polymerase chain reaction
IQR	interquartile range
PEG	pegylated
HR	hazard ratio
CI	confidence interval
EVs	esophageal varices
GVs	gastric varices
